# 2'-Hydroxyflavanone: A novel strategy for targeting breast cancer

**DOI:** 10.18632/oncotarget.20499

**Published:** 2017-08-24

**Authors:** Jyotsana Singhal, Lokesh Nagaprashantha, Shireen Chikara, Sanjay Awasthi, David Horne, Sharad S. Singhal

**Affiliations:** ^1^ Department of Molecular Medicine, Beckman Research Institute of City of Hope, Comprehensive Cancer Center and National Medical Center, Duarte, CA 91010, USA; ^2^ Department of Medical Oncology, Texas Tech University Health Sciences Center, Lubbock, TX 79430, USA

**Keywords:** breast cancer, 2'-hydroxyflavanone, RLIP76, VEGF, xenografts

## Abstract

Breast cancer is the most common cancer in women that is driven by cross-talk with hormonal and cellular signaling pathways. The natural phytochemicals, due to broad-spectrum anti-inflammatory and anti-cancerous properties, present with novel opportunities for targeting breast cancer. Intake of citrus fruits is known to reduce the risk for incidence of breast cancer. Hence, we tested the efficacy of citrus flavonoid 2'-hydroxyflavanone (2HF) in breast cancer. 2HF inhibited survival, clonogenic ability, cell cycle progression and induced apoptosis in breast cancer cells. 2HF also decreased VEGF levels and inhibited migratory capacity of breast cancer cells. Administration of 2HF led to regression of triple-negative MDA-MB-231 tumors in the mice xenograft model. 2HF decreased the levels of RLIP76 both *in vitro* studies and *in vivo* MDA-MB-231 xenograft model of breast cancer. Western blot and histopathological analyses of resected tumors showed a decline in the levels of survival and proliferation markers Ki67, pAkt, survivin, and cell cycle proteins CDK4 and cyclin B1. 2HF treatment led to inhibition of angiogenesis as determined by decreased VEGF levels *in vitro* and angiogenesis marker CD31 *in vivo*. 2HF reversed the pro-/anti-apoptotic ratio of BAX/BCL-2 by decreasing anti-apoptotic protein BCL-2 and increasing pro-apoptotic proteins BAX and BIM *in vivo*. 2HF also decreased the mesenchymal markers vimentin and fibronectin along with causing a parallel increase in pro-differentiation protein E-cadherin. Collectively, the ability of 2HF to decrease RLIP76, VEGF and regulate critical proliferative, apoptotic and differentiation proteins together provides strong rationale to further develop 2HF based interventions for targeting breast cancer.

## INTRODUCTION

Breast cancer remains a leading cause of cancer-related deaths among women worldwide despite significant advances in targeted therapies and screening techniques for early detection [1–4]. Breast cancer is a very aggressive tumor with notoriously poor prognosis following metastases [5]. The known risk factors for the onset of breast cancer include family history, Li-Fraumeni syndrome, atypical hyperplasia of the breast, late-age at first full-term pregnancy, early menarche, and late menopause [6–8]. Selective estrogen-receptor (ER) modulators (e.g., tamoxifen) appear promising for prevention of breast cancer; however, they are largely ineffective against ER-negative breast cancers [9, 10]. Moreover, selective ER modulators have serious side effects such as increased risk of uterine cancer, thromboembolism, cataracts, and perimenopausal symptoms [10, 11]. Triple negative breast cancer (TNBC), characterized by the absence of ER, progesterone receptor (PR) and lacking in *HER2* gene expression, is associated with more aggressive phenotype [12]. TNBC is more prevalent in younger females and accounts for about 10-20% of breast cancers with characteristic poor prognosis and low survival rates [5]. In addition, about 25-46% patients with TNBC are at higher risk of brain metastasis [13]. Therefore, novel strategies for targeting breast cancers, irrespective of hormone receptor and HER2 status, are highly desirable.

RLIP76 (a 76 kDa ral-binding protein, RALBP1 or RLIP76) is a multi-functional rac and ral effector that also functions as a major and multi-specific glutathione-conjugate (GS-E) transporter of mercapturic acid pathway (MAP) [14]. The knockout of RLIP76 leads to inhibition of epithelial carcinogenesis [15]. RLIP76 is up-regulated in multiple cancers and is known to induce apoptotic- and drug-resistance by mediating active efflux of the GS-Es of chemotherapy drugs and toxic products of lipid peroxidation [14–22]. RLIP76 also regulates the endocytosis of receptor-ligand complexes in IGF and EGFR signaling [15]. RLIP76 has been shown to be an essential factor in determining the proliferative potential and metastatic signaling of cancers [16–22]. Due to its multiple regulatory effects on the incidence, proliferation, invasion and metastases of cancers, RLIP76 represents a vital target for breast cancer.

Natural phytochemicals have received increasing attention in recent years for the discovery of anticancer agents [23]. Flavonoids, a large group of polyphenolic compounds present in foods and beverages of plant origin, have anti-oxidant, anti-inflammatory, anti-mutagenic, and anti-proliferative properties [24–26]. The intake of citrus fruits and citrus juice has been associated with protection from breast cancer incidence in both animal models and in humans [27, 28]. Hence, we tested the effect of citrus flavonoid 2'-Hydroxyflavanone (2HF) *in vitro* and *in vivo* on the survival and progression of breast cancer. MCF-7 breast cancer cells are known to be ER^+^, PR^+^ and HER2^-^ while MDA-MB-231 cells are known to have characteristics of ER^-^, PR^-^ and HER2^-^ (triple-negative) breast cancers [29]. In the present study, we observed that 2HF treatment inhibits the growth of breast cancer cells *in vitro*, reduces the levels of RLIP76 and VEGF, and inhibits the progression of triple-negative MDA-MB-231 breast tumors in xenograft mouse model of breast cancer. The results from our collective *in vitro* and *in vivo* investigations elucidated the anticancer potential of 2HF as a novel small molecule candidate drug for targeting breast cancer.

## RESULTS

### 2HF inhibits proliferation and colony forming ability in breast cancer cells *in vitro*

**Figure 1:**
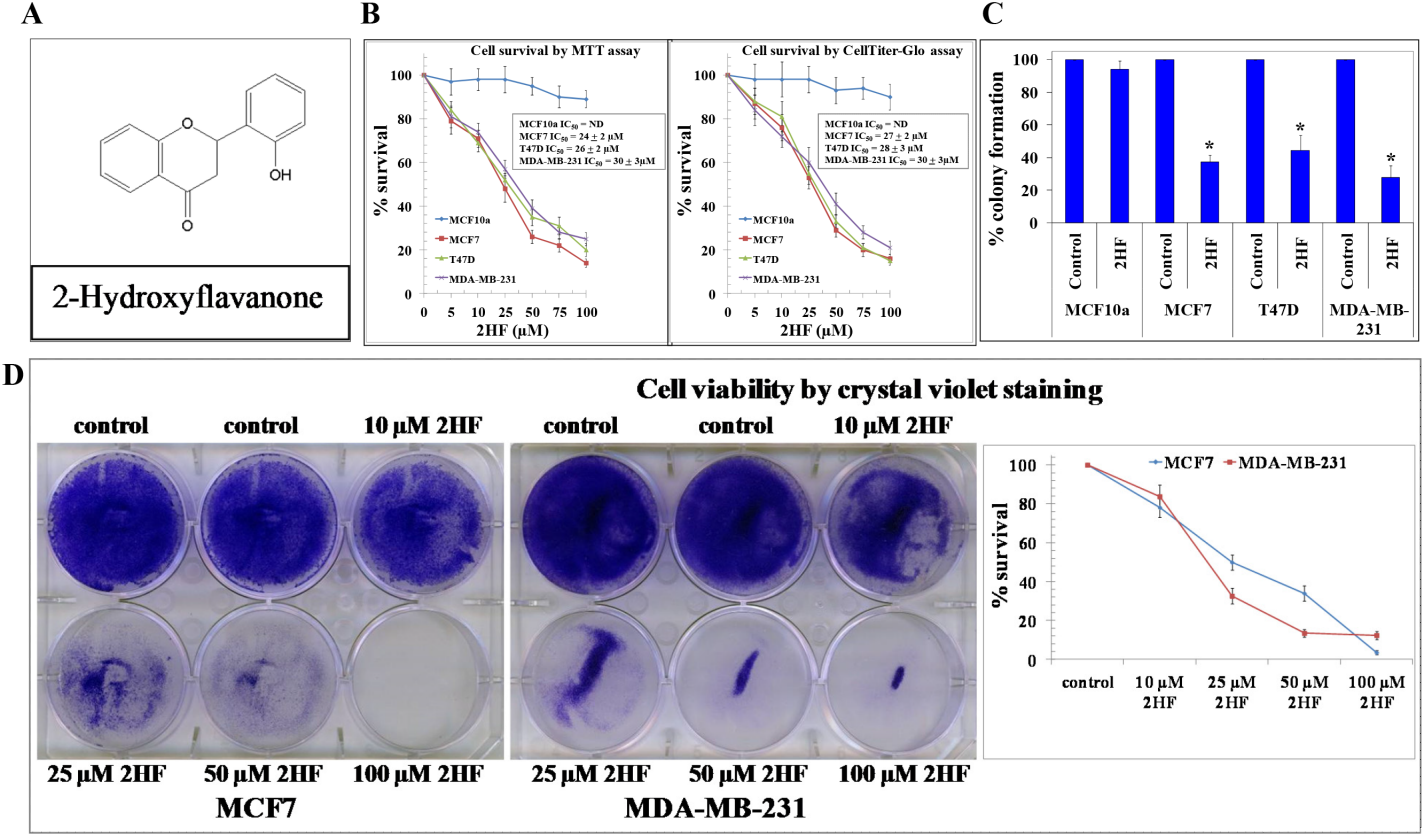
2HF inhibits breast cancer cell survival sparing normal breast epithelial cells The chemical structure of 2HF **(panel A)**. Drug-sensitivity assays were performed by MTT as well as CellTiter Glo assay using 2HF at 48 h post treatment to determine IC_50_. Values are presented as mean ± SD from three separate determinations with 8 replicates each (n = 24) **(panel B)**. Colony-forming assay was performed and the colonies were counted using Innotech Alpha Imager HP. The bars represent the % colony formation (*p < 0.001 compared with Control) **(panel C)**. Cells were treated with various concentrations of 2HF. After a 48 h exposure, cell viability was assessed by crystal violet staining. Values are presented as mean ± SD from three separate experiments as compared with the Control (n = 3) **(panel D)**.

2HF is a novel phytochemical that is known for anti-inflammatory and anticancer properties [24–26]. The structure of 2HF is represented in Figure 1A. We conducted *in vitro* studies to assess the impact of 2HF on cell survival, clonogenic ability and cytotoxicity. MTT assay conducted following 48 h of 2HF treatment revealed that 2HF is effective in inhibiting survival of MCF-7 as well as aggressive MDA-MB-231 and T47D breast cancer cell lines with an IC_50_ ranging from 24±2 to 30±3 μM (Figure 1B, Table 1A) [30]. The CellTiter-Glo luminescent assay also confirmed inhibitory effects of 2HF in breast cancer cells with an IC_50_ ranging from 27±2 to 30±3 μM (Figure 1B, Table 1A) [31]. The 50 μM of 2HF treatment caused ∼ 45-75% inhibition of colony forming ability of MCF-7, T47D, and MDA-MB-231 breast cancer cell lines (Figure 1C, Table 1B). In addition, crystal violet staining revealed decrease in viable cells following 2HF treatment (Figure 1D). Interestingly, MCF10A normal human mammary epithelial cells were significantly more resistant to 2HF-mediated anti-proliferative effects as compared with MCF-7, MDA-MB-231 and T47D breast cancer cells (Figure 1).

**Table 1A:** IC_50_ **(µM) values in breast cancer cells following 2HF treatment**

Cell Line	MTT Assay	CellTiter-Glo Assay
MCF10a	ND	ND
MCF-7	24±2	27±2
T47D	26±2	28±3
MDA-MB-231	30±3	30±3

NOTE: Drug sensitivity assays were performed following 2HF treatment for 48 h in both MTT and CellTiter-Glo assays. ND: Not detected. Values represent mean ± SD from three separate determinations with eight replicates (*n*=24).

**Table 1B:** Colony formation assay following 2HF treatment in breast cancer cells

Cell Line	% Colony formation (as compared to Controls)	SD
MCF10a	94	5
MCF-7	37	4
T47D	44	9
MDA-MB-231	28	7

NOTE: Values represent mean ± SD from three separate determinations with triplicates (n=9).

### 2HF induces apoptosis, inhibits migratory capacity, decreases RLIP76 and VEGF, and induces cell cycle arrest in breast cancer cells *in vitro*

**Figure 2:**
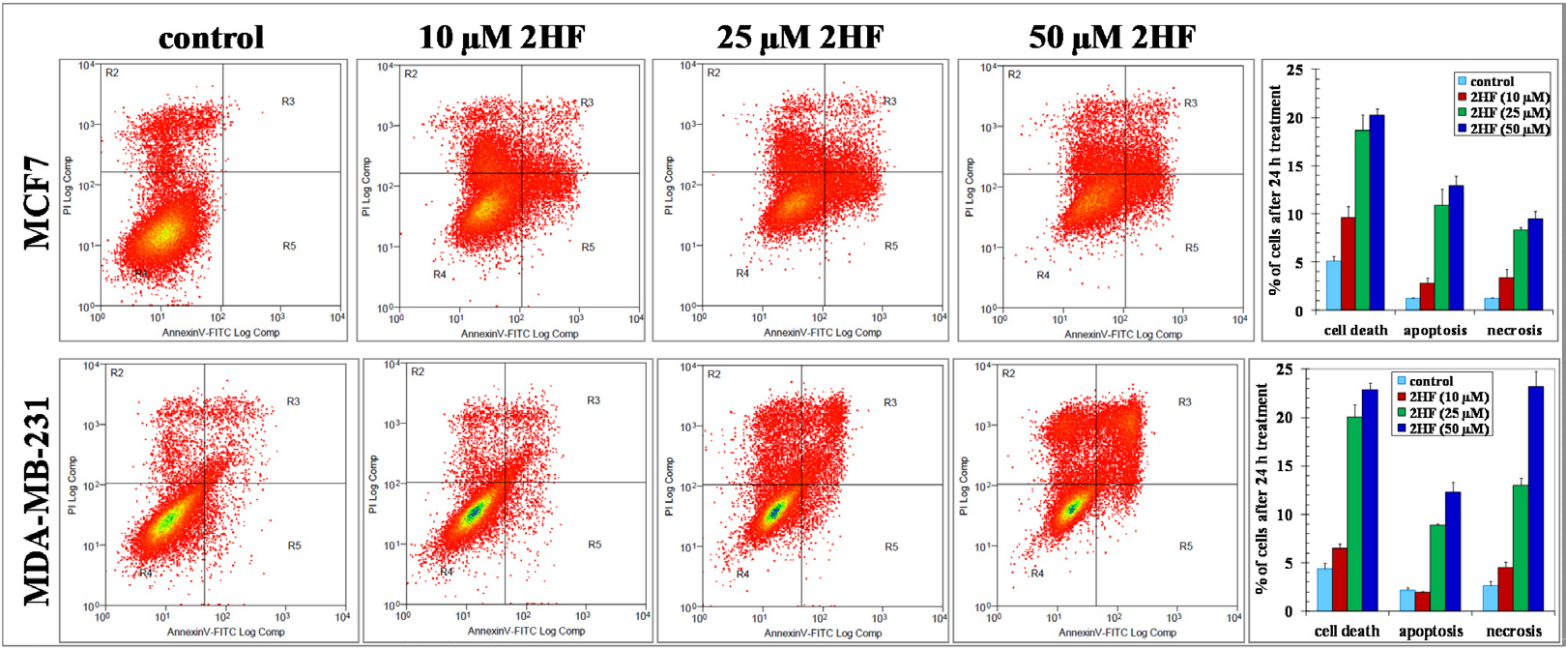
2HF induces apoptosis as assessed by Annexin V Assay MCF7 and MDA-MB-231 cells were incubated with or without test compound for 24 h, washed and harvested. The cells were then fixed and double stained with Annexin V-FITC and PI, and analyzed by flow cytometry. The percentage distribution of normal/viable, early apoptotic, late apoptotic, and necrotic cells was calculated using Summit software. The experiment was repeated three times and similar results were obtained. *p<0.001, compared with Control.

**Figure 3:**
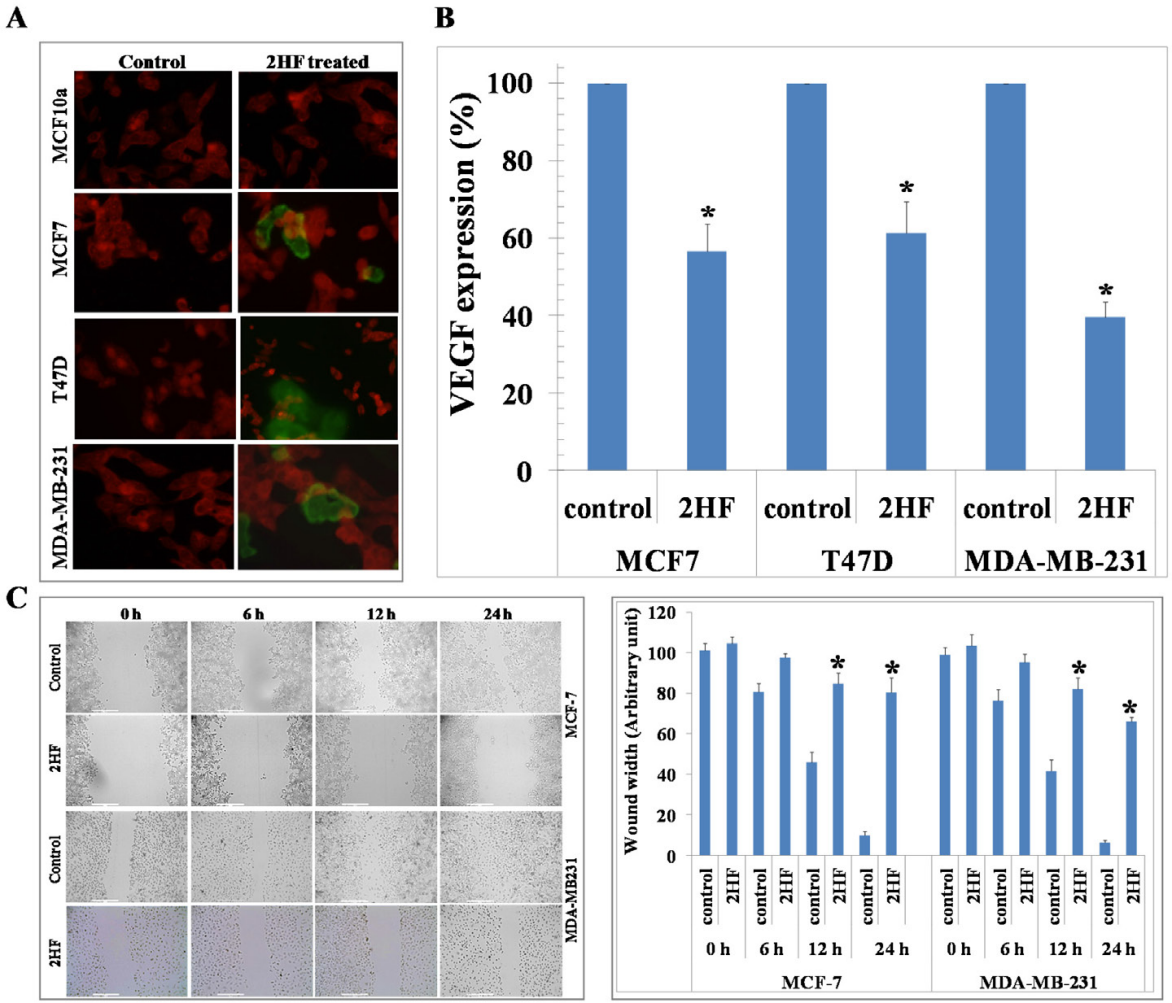
2HF induces apoptosis as assessed by TUNEL assay, decreases VEGF and inhibits migration of breast cancer cells Normal (MCF10a) and breast cancer (MCF-7, T47D, MDA-MB-231) cells were grown on cover-slips. For 2HF treatment, cells were incubated with 50 μM 2HF for 24 h before TUNEL assay using Promega fluorescence detection kit according to the protocol provided by the manufacturer. Slides were analyzed by laser scanning fluorescence microscope (Zeiss LSM 510 META). Photographs taken at identical exposure at 400 x magnification are presented. Apoptotic cells showed green fluorescence **(panel A)**. VEGF expression in Control and 2HF-treated cells by enzyme-linked immunosorbent assay kit (R&D System): *p < 0.01 compared with Control **(panel B)**. 2HF treatment inhibited migration of cultured MCF-7 and MDA-MB-231cells:MCF-7 and MDA-MB-231 cells were grown in 6-well plates to form monolayer. Wounds were created using 0.1 ml sterile tip. Cell migration capacity was measured by the time taken to heal the wound in Control and 2HF-treated cells. Representative images depicting the effect of 2HF treatment on MCF-7 and MDA-MB-231 cells migration. Significantly different (*p<0.001) compared with Control by Student’s *t* test **(panel C)**.

2HF-induced apoptotic effects were initially assessed by Annexin V assay which showed a concentration dependent induction of apoptosis in both MCF-7 and MDA-MB-231 breast cancer cells (Figure 2). A 50 μM treatment of 2HF for 24 h induced apoptosis in MCF-7, MDA-MB-231 and T47D breast cancer cells, while sparing normal MCF10A cells as determined by DNA fragmentation in TUNEL apoptosis assay (Figure 3A). The levels of VEGF and the ability of cancer cells to migrate determine their ability to form blood vessels and metastasize [32]. 2HF treatment lead to ∼40-60% inhibition of VEGF levels with a marked anti-VEGF effect in aggressive MDA-MB-231 cells as compared to MCF-7 and T47D cells (Figure 3B). Wound healing assay at 6 h, 12 h, and 24 h showed that 2HF treatment inhibits the migratory capacity of MCF-7 and MDA-MB-231 breast cancer cells (Figure 3C).

**Figure 4:**
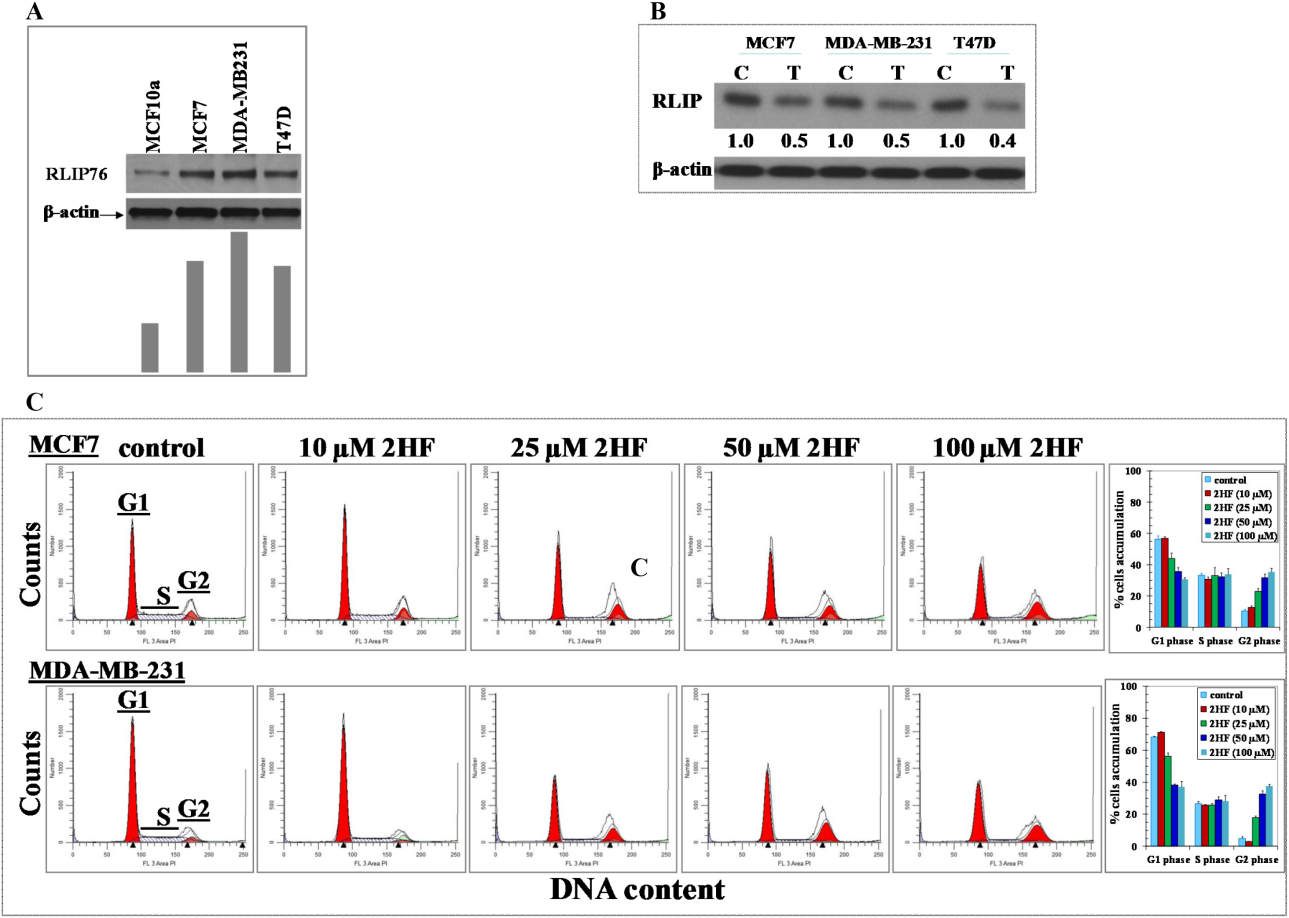
2HF decreases RLIP76 protein levels and inhibits cell cycle progression in human breast cancer cells Aliquots of crude membrane extracts of respective cells were analyzed by Western blot analyses against anti-RLIP76 IgG. Results were quantified by scanning densitometry. β-actin was used as an internal control **(panel A)**. Western blots show the change in cellular RLIP76 protein content upon treatment of the human breast cancer cell lines with 25 μM 2HF for 24 h. β-actin was used as a loading control. Numbers below the blots represent the fold change in the level of RLIP76 as compared to Control as determined by densitometry **(panel B)**. Inhibitory effect of 2HF on cell cycle distribution was determined by fluorescence activated cell sorting (FACS) analysis. Experimental details are given in the *Methods section*. The stained cells were analyzed using a CyAn ADP cytometer (Beckman Coulter Inc) **(panel C)**. The experiment was repeated three times and similar results were obtained, *p<0.001, compared with Control.

RLIP76 is a critical protein essential for migration, survival, and proliferation of cancer cells [14–22]. We further assessed the endogenous levels of RLIP76 in breast cancer cells and the effect of 2HF on the levels of RLIP76 protein expression in breast cancer cells. Western blot analyses revealed an elevated level of RLIP76 in MCF-7, MDA-MB-231 and T47D cells (Figure 4A). The treatment of MCF-7, MDA-MB-231 and T47D cells with 25 μM of 2HF for 24 h led to ∼50-60% decrease in the levels of RLIP76 protein in all the treated cell lines (Figure 4B). This finding denotes that inhibition of RLIP76 protein expression is a common mechanism, along with inhibition of VEGF, by which the anticancer effects of 2HF are mediated in breast cancer cells with diverse ER, PR and HER2 status (Figure 3B and 4B). The anti-proliferative effects of 2HF were further examined by cell cycle FACS analysis. 2HF treatment caused G2/M phase arrest in MCF-7 and MDA-MB-231 human breast cancer cells with ∼40% cells accumulated in G2 phase (Figure 4C). Following the assessment of *in vitro* anticancer properties of 2HF in MCF-7, MDA-MB-231 and T47D breast cancer cells, we further investigated the efficacy of 2HF in mice xenograft model established using aggressive MDA-MB-231 breast cancer cells.

### 2HF administration inhibits the growth of MDA-MB-231 xenografts in nude mice

**Figure 5:**
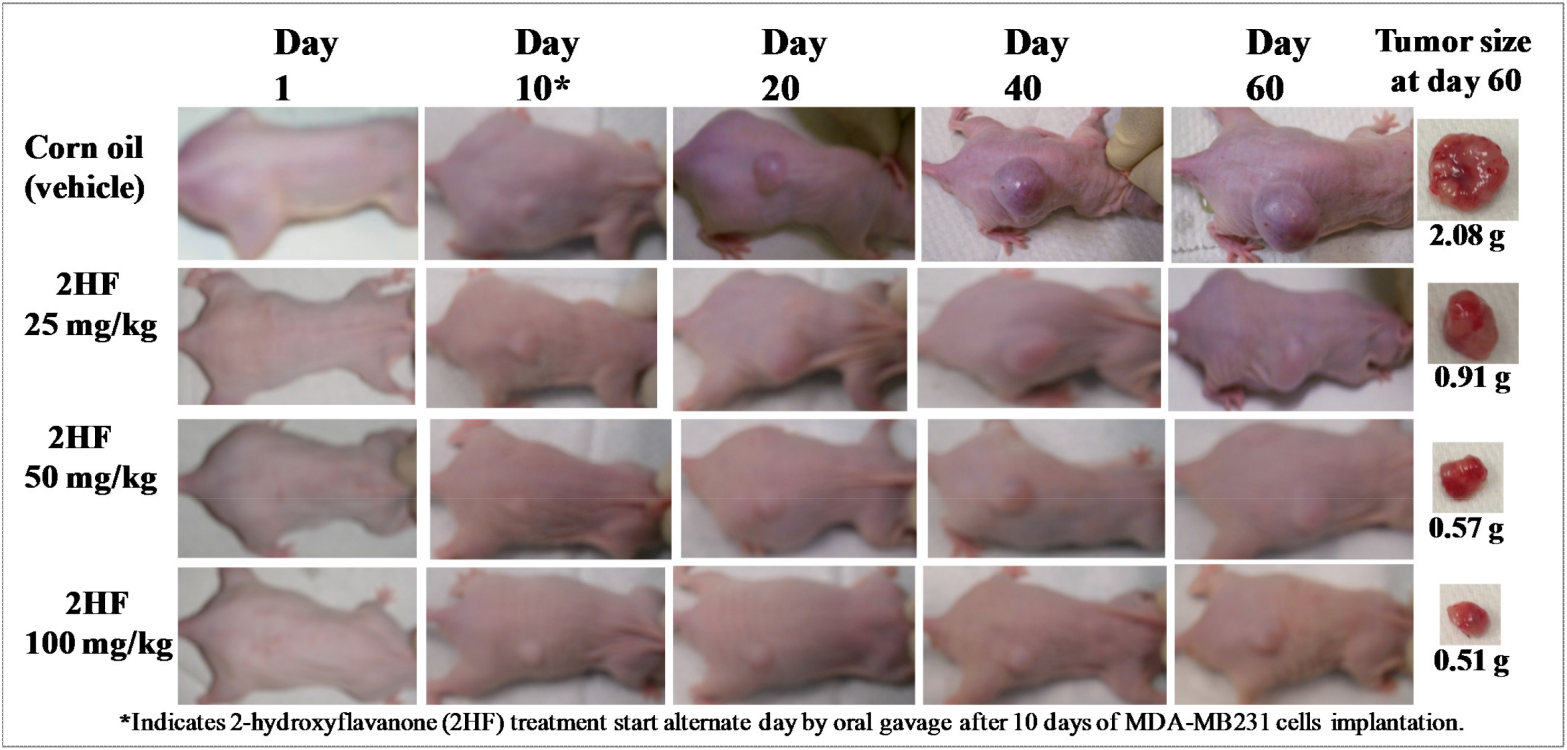
2HF inhibits progression of triple-negative MDA-MB-231 breast xenograft tumors in nude mice Hsd: Athymic 8 wk old female nude nu/nu mice were obtained from Charles River, Wilmington, MA, and acclimated for a week prior to start of the experiment. All animal experiments were carried out in accordance with a protocol approved by the Institutional Animal Care and Use Committee (IACUC). Twenty mice were divided into four groups of 5 animals (treated with corn oil (vehicle), and 2HF 25, 50 and 100 mg / kg b.w.). MDA-MB231 cells were suspended in PBS and mixed in a 1:1 ratio with Matrigel. All 20 animals were injected with 1 x 10^6^ breast cancer cells (MDA-MB-231) suspensions in 100 μl of PBS, subcutaneously into one flank of each nu/nu nude mouse. At the same time, animals were randomized treatment groups as indicated in the figure. Treatment was started 10 days after the MDA-MB-231 cells implantation to see palpable tumor growth. Treatment consisted of 25, 50 and 100 mg/kg b.w. of 2HF in 200 μl corn oil by oral gavage alternate day. Control groups were treated with 200 μl corn oil by oral gavage alternate day. Animals were examined daily for signs of tumor growth. Tumors were measured in two dimensions using calipers and body weights were recorded. Photographs of animals were taken at day 1, day 10, day 20, day 40, and day 60 after subcutaneous injection, are shown for all groups. Photographs of tumors were also taken at day 60.

**Figure 6:**
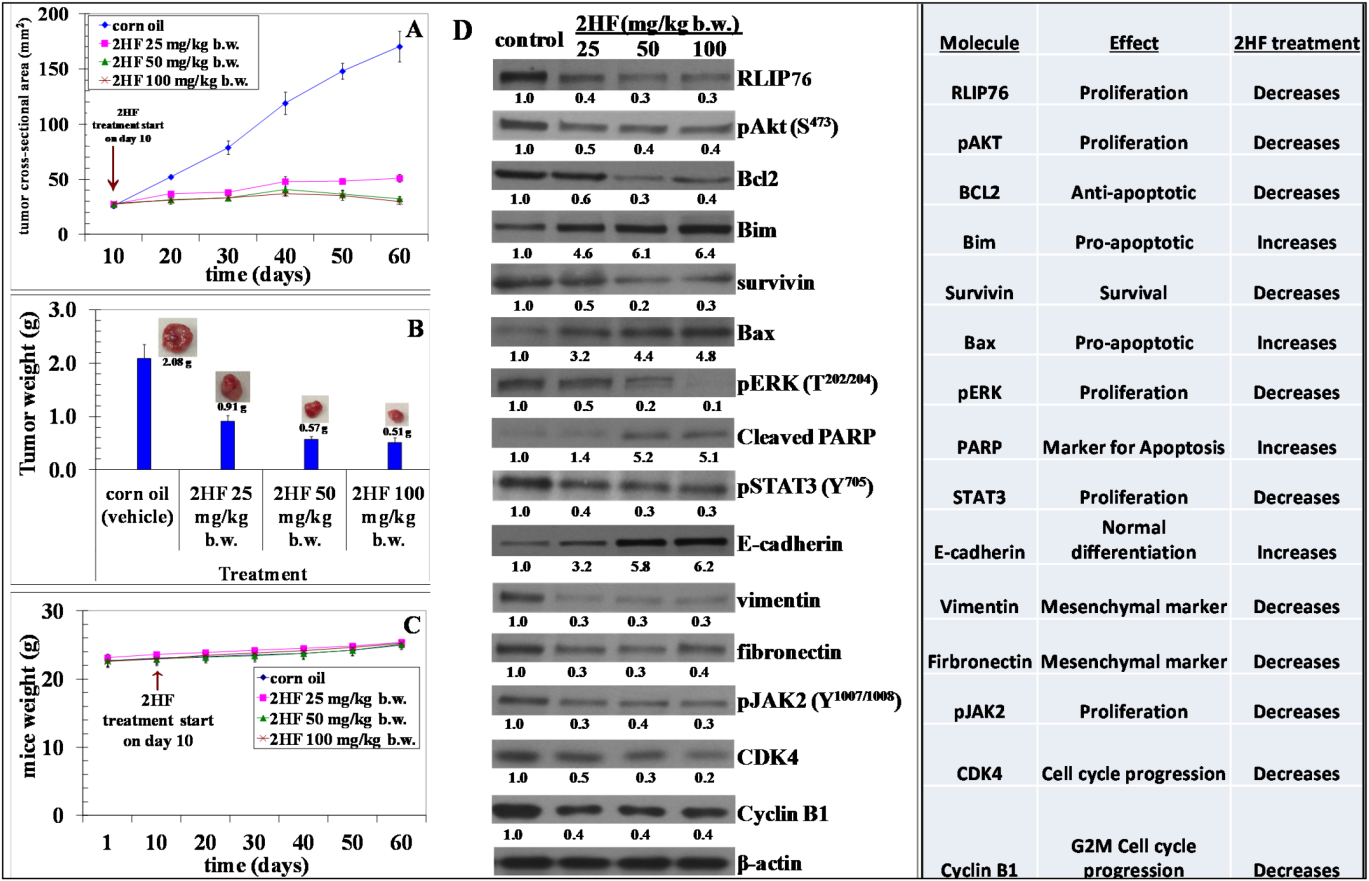
2HF decreases final tumor weight and regulates the levels of critical tumor proteins in MDA-MB-231 breast xenograft tumors Effect of 2HF on tumor cross sectional area: Tumors were measured by calipers **(panel A)**. Effect of 2HF on tumor weights: Tumors were excised on day 60 after implantation and weighed before use for immuno-histochemical experiments. The weight of 2HF-treated tumors was found to be statistically different from Controls (corn oil treated) (*p* < 0.005) **(panel B)**. Tumor-bearing mice were weighed over the course of the experiment as a measure of possible 2HF toxicity **(panel C)**. Effect on cancer signaling pathways in excised tumors from breast cancer xenograft after treatment with 2HF: Western blot analyses of signaling proteins in MDA-MB-231 human breast cancer tumor tissue lysates in Control and 2HF treated experimental groups. β-actin was used as a loading control. Numbers below the blots represent the fold change in the levels of proteins as compared to Control as determined by densitometry **(panels D)**.

For breast cancer xenograft studies, a 0.1 mL suspension containing 1x10^6^ MDA-MB-231 cells was injected subcutaneously on right flank of female nude mice above the hind limb. Treatment was started 10 days after MDA-MB-231 cells were implanted, a time point when palpable tumor growth was seen in our mice xenograft model. Photographs of animals were taken at day 1, day 10, day 20, day 40, and day 60 after subcutaneous injection, and are shown for all groups (Figure 5). Tumors grew more slowly in mice xenografts administered with 2HF than in respective untreated control mice. During the course of study, the average tumor size in mice treated with 25, 50, and 100 mg / kg b.w. 2HF was significantly lower when compared with vehicle-treated controls. Our *in-vivo* studies showed that oral administration of 2HF to nude mice bearing aggressive MDA-MB-231 breast cancer xenografts leads to significant inhibition of tumor progression in 2HF treated groups with uncontrolled tumor growth observed in the animals treated with vehicle only (*p*<0.002; Figure 6A). At day 60 following tumor cell injection, the average tumor size in control mice was about 3.3- to 5.6-fold higher as compared to average tumor size of 2HF-treated mice. The average wet weight of the tumor at day 60 was also lower in the 2HF-treated groups [2HF (25 mg / kg b.w.): 0.91g, 2HF (50 mg / kg b.w.): 0.57g. 2HF (100 mg / kg b.w.): 0.51g] as compared with control mice [2.08g] (Figures 5 and 6B). In addition, the 2HF-treated mice did not exhibit any signs of stress such as impaired movement or posture, indigestion, and areas of redness or swelling. The initial and the final body weights of the control and 2HF-treated mice did not differ significantly (Figure 6C). These results provide critical *in vivo* evidence for the growth inhibitory effects of 2HF in breast cancer.

### 2HF regulates the levels of critical tumor proteins *in-vivo*

**Figure 7:**
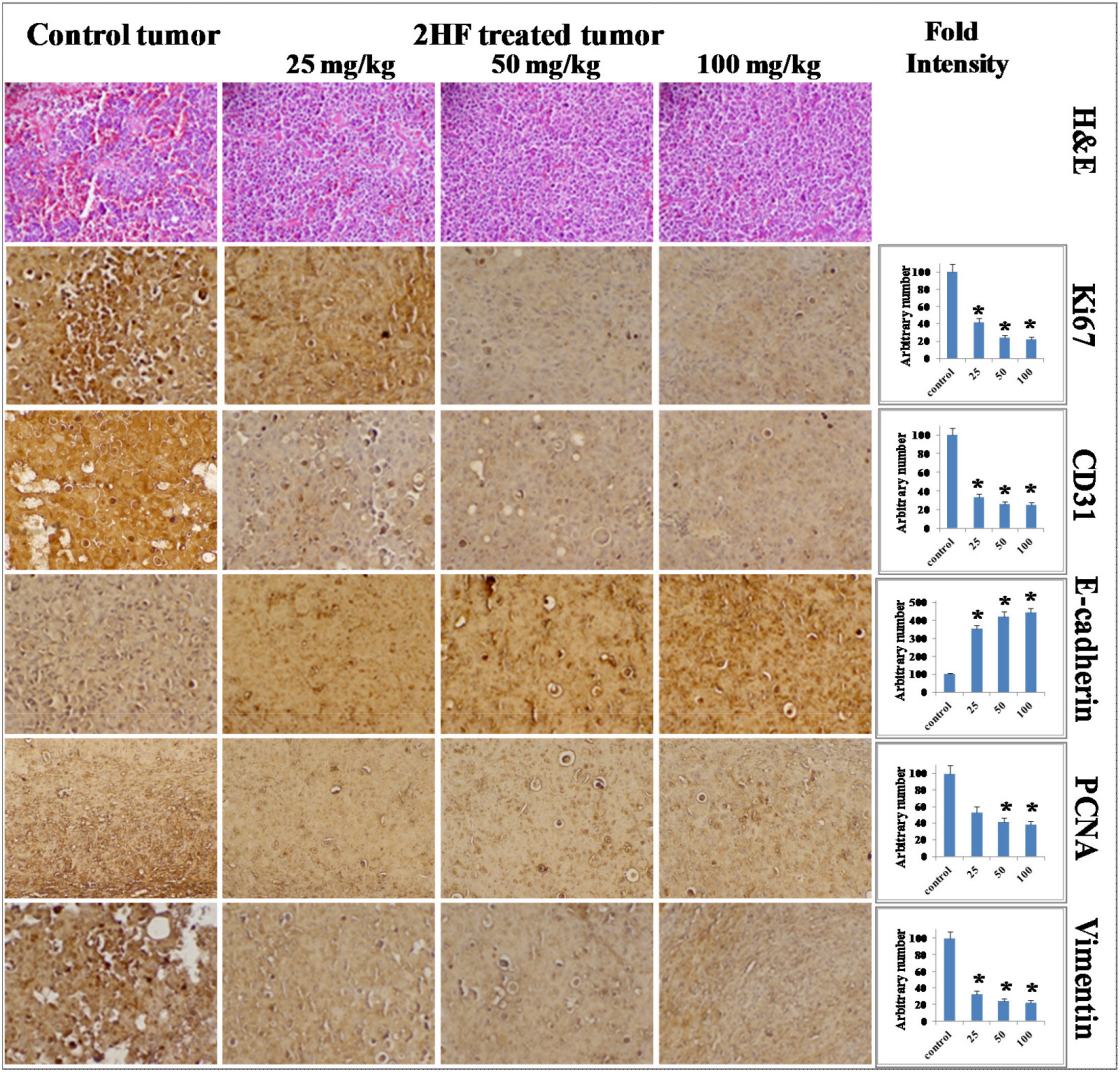
Immunohistochemical analyses revealing that 2HF inhibits proliferation and angiogenesis markers in MDA-MB-231 breast xenograft tumors Control and 2HF-treated breast cancer bearing nude mice tumor sections were used for histopathologic analyses. Presented are H & E stained sections, IHC analyses for the expression of Ki67, CD31, E-cadherin, PCNA, and vimentin. Statistical significance of difference was determined by two-tailed Student’s *t* test. *p* < 0.001, 2HF-treated compared with Control. Photomicrographs at 40x magnification were acquired using Olympus DP 72 microscope. Percent staining was determined by measuring positive immuno-reactivity per unit area. The intensity of antigen staining was quantified by digital image analysis using Pro Plus software. Bars represent mean ± S.E. (n = 5).

Tumor tissue lysates from 2HF treated and control MDA-MB-231 mice xenografts were assessed for proliferative, apoptotic and cell cycle markers by Western blot and histopathology. The Western blot analyses of 2HF treated tumor tissue lysates, as compared with untreated controls, revealed a decrease in the level of proliferation proteins pAKT, survivin, RLIP76, pERK, pJAK2, STAT3, CDK4 and cyclin B1 (Figure 6D). The 2HF treated tumor tissues also showed a decrease in anti-apoptotic protein BCL-2 and mesenchymal markers vimentin and fibronectin. The Western blot analyses also revealed that the pro-apoptotic proteins BAX and BIM, apoptosis marker PARP, and normal differentiation marker E-cadherin were increased in 2HF-treated tumor tissue lysates as compared to untreated controls (Figure 6D). The immunohistochemical (IHC) analyses of 2HF treated breast tumors showed decreased levels of proliferation markers Ki67 and PCNA, angiogenesis marker CD31, and increased levels of epithelial differentiation marker E-cadherin (Figure 7) [33].

Taken together, our results indicate that 2HF displays strong anticancer effects in breast cancer. 2HF is a novel natural small phytochemical that can decrease the levels of RLIP76 in breast cancer cells. The efficacy of 2HF in aggressive, triple-negative MDA-MB-231 breast xenograft tumors along with observed effects on critical survival, apoptotic, angiogenic and differentiation proteins further provides both efficacy and mechanistic basis for the anticancer potential of 2HF in breast cancer.

## DISCUSSION

Effective management of aggressive breast tumors remains a challenge in healthcare. The breast tumors arising from epithelial and basal regions of mammary gland display different characteristics and necessitate different approaches [4]. In addition, the variations in the response to conventional therapies based on hormone receptor status make breast tumors a challenging entity for management [2, 5]. Flavonoids are compounds ubiquitously present in many food sources. These naturally occurring compounds have been linked to multiple biologic functions, such as anti-proliferative, pro-apoptotic, anti-inflammatory, anti-invasive and anti-angiogenic effects [34, 35]. 2HF is a novel natural small phytochemical with no overt toxicity towards normal tissues [26]. In the present study, we investigated the molecular mechanisms of 2HF-induced anticancer effects in breast cancer. The treatment of 2HF led to potent cytotoxic effects as confirmed by multiple cell survival, proliferation and clonogenic assays. 2HF induced apoptosis and cell cycle inhibition was evident in both ER^+^ and ER^-^ breast cancer cells.

We also observed that the endogenous protein levels of RLIP76 were increased in breast cancer cells. Hence, we investigated the effect of 2HF on the levels of RLIP76. 2HF was effective in decreasing RLIP76 protein levels in breast cancer cells irrespective of ER, PR and HER2 status. RLIP76 is a multifunctional protein which primarily serves as the transporter of glutathione-conjugates (GS-E) of products of lipid peroxidation and chemotherapy drugs, thereby reducing the effective intracellular concentrations of toxic products in cancer cells [36–38]. RLIP76 serves as a rate-limiting step for ligand-receptor endocytosis in cell membrane and regulates the downstream tyrosine kinase signaling that impacts proliferation, survival and apoptosis [14, 15]. The inhibition and/or depletion of RLIP76 is known to exert potent anti-proliferative effects in cancers of lung, colon, prostate, kidney, neuroblastoma and melanoma [16-18, 20, 21].The *in vitro* results when taken together with the ability of 2HF to inhibit the tumor progression and decrease RLIP76 protein levels *in vivo* triple-negative MDA-MB-231 breast cancer xenografts further provides evidence for the role of RLIP76 in mediating the effects of 2HF in breast cancer.

It is possible that 2HF could be effective in breast cancer by multiple mechanisms. Several findings in this study are mechanistically novel and translationally significant for targeting aggressive breast cancers. First, the down-regulation of RLIP76 and VEGF consequent to 2HF treatment is a major and significant finding for both prevention and for targeting aggressive types of breast cancer. RLIP76, along with being a critical regulator of cellular tyrosine kinase signaling cascades, is essential for angiogenesis in tumors [14, 15, 39]. Angiogenesis is especially critical for growth and progression of solid tumors since growth in tumor mass beyond 2-3 mm is often preceded by an increase in formation of new blood vessels presumably essential for delivery of nutrients and oxygen to the tumor microenvironment [40, 41]. We initially found that *in vitro* exposure of breast cancer cells to 2HF results in the suppression of VEGF levels (Figure 3B). In addition, the present study indicates that the 2HF-mediated suppression of MDA-MB-231 xenograft growth is accompanied by inhibition of neovascularization in the tumor as evidenced by a marked reduction in the vessel area and decrease in CD31, a marker of angiogenesis. This may imply a potential for combination treatments with 2HF and biologic/or chemotherapeutic agents. Second, we observed a reversal of the pro-/anti-apoptotic ratio of BAX/BCL-2 [42]. Our experiments demonstrate a 2HF-induced down-regulation of anti-apoptotic BCL-2 which is paralleled by an increase in pro-apoptotic BAX thereby producing an apoptotic response in MDA-MB-231 xenografts. The pJAK2 and its downstream target STAT3, which regulates BCL-2 and survivin levels, were also decreased following 2HF treatment [43]. Third, reduction of cyclin B1 and CDK4 further confirmed the observed effects of 2HF on cell cycle. Fourth, epithelial-to-mesenchymal transformation (EMT) leads to increased capacity for survival, invasion and metastatic colonization of tumor cells. In our immunohistochemical analyses, the tumor levels of pro-differentiation marker E-cadherin was increased by 2HF exposure, which was paralleled by a decrease in EMT markers vimentin and fibronectin. These findings signalize a reversal of immuno-phenotypic changes, paradigmatic for the EMT that can otherwise trigger aggressive tumor growth [44].

Further studies are needed to explore the bioavailability of 2HF on acute and chronic administration to derive evidence on the dosing of 2HF for breast cancer prevention and therapy. In conclusion, the ability of 2HF to decrease the levels of RLIP76 and VEGF as observed by both *in vitro* and *in vivo* studies and the observed impact on the regulation of critical markers of breast cancer growth, angiogenesis, invasion and therapeutic sensitivity together provide a strong basis for further characterization and development of 2HF for targeting breast cancer.

## MATERIALS AND METHODS

### Ethics statement

No human subjects are involved in the present study. All the animal studies were conducted in following City of Hope Animal Care and Ethics Committee approval according to IACUC protocol # 12024.

### Materials

2HF (purity ∼99%), Horseradish peroxidase (HRP)-conjugated anti-mouse, and anti-rabbit secondary antibodies, and MTT were purchased from Sigma-Aldrich, St. Louis, MO, USA. The CD31, Ki67, ERα, cyclin B1, CDK4, BCL-2, survivin, BIM, BAX, pAkt (S^473^), pERK (T^202/204^), pSTAT3 (Y^705^), cleaved poly-ADP ribose polymerase (PARP), pJAK2 (Y^1007/1008^), vimentin, fibronectin, and E-cadherin antibodies were purchased from Santa Cruz Biotechnology (Columbus, OH, USA) and Cell Signaling Technologies (Danvers, MA, USA). ELISA kit for VEGF expression was procured from R & D Systems (Minneapolis, MN, USA). TUNEL fluorescence and CellTiter-Glo were procured from Promega (Madison, WI, USA). Matrigel was purchased from Corning Life Sciences (Tewksbury, MA, USA). Avidin/biotin complex (ABC) detection kit was procured from Vector (Burlingame, CA, USA). The universal *Mycoplasma* detection kit was procured from ATCC (Manassas, VA, USA).

### Cell lines and cultures

Human breast untransformed (MCF10a) and cancer (MCF-7, T47D and MDA-MB-231) cell lines were purchased from the American Type Culture Collection (ATCC, Manassas, VA, USA). The authentication of cell lines was done by analyzing fifteen different human short tandem repeat (STR) at Genomic Core, Beckman Research Institute of City of Hope, Duarte, CA, to test for interspecies contamination. The cell lines were last tested in March 2017. All cells were cultured at 37 °C in a humidified atmosphere of 5 % CO_2_ in the appropriate medium: DMEM/F12 with 15 mM Hepes buffer, 5% horse serum, 10 μg/ml insulin, 20 ng/ml EGF, 100 ng/ml cholera toxin, 0.5 μg/ml hydrocortisone (MCF10a), RPMI-1640 (MCF-7, T47D) and DMEM (MDA-MB-231), medium supplemented with 10% fetal bovine serum (FBS) and 1% penicillin/streptomycin (P/S) solution. All the cells were also tested for Mycoplasma once every 3 months.

### Drug sensitivity (MTT) assay

Cell density measurements were performed using a hemocytometer to count viable cells resistant to staining with trypan blue. Approximately 20,000 cells were plated into each well of 96-well flat-bottomed micro-titer plates. After 12 h incubation at 37^°^C, medium containing 2HF (ranging 0-100 μM) were added to the cells. After 48 h incubation, 20 μl of 5 mg/ml MTT were introduced to each well and incubated for 2 h. The plates were centrifuged and medium was decanted. Cells were subsequently dissolved in 100 μl DMSO with gentle shaking for 2 h at room temperature, followed by measurement of OD_570_. Eight replicate wells were used at each point in each of three separate measurements [45–47].

### CellTiter-Glo luminescent cell viability assay

Approximately 10,000 cells were plated into each well of 96-well flat-bottomed micro-titer plates. After 12 h incubation at 37^°^C, medium containing 2HF (ranging 0-100 μM) were added to the cells. After 48 h incubation at 37^°^C, the 96-well plate out of the incubator and equilibrate the plate at room temperature for 5 min. Discard the culture media, washed with PBS and add 50 μl of PBS in each well. Add 50 μl of CellTiter-Glo to each well. Mix contents for 2 min on an orbital shaker to induce cell lysis, and allow the plate to incubate at room temperature for 10 min to stabilize luminescent signal. Transfer the content from each well of the 96-well plate to a new opaque-walled multi-well plate. Prepared control wells containing PBS without cells and CellTiter-Glo to obtain a value for background, and luminescence was recorded. Eight replicate wells were used at each point in each of three separate measurements [47].

### Colony formation assay

Cell survival was evaluated using a standard colony-forming assay. 1x10^5^ cells / ml were incubated with 2HF (50 μM) for 24 h, and aliquots of 50 or 100 μl were added to 60-mm size tissue culture dishes containing 4 ml culture medium. After 10 days, adherent colonies were fixed, stained with 0.5% methylene blue for 30 min, and colonies were counted using the Innotech Alpha Imager HP [45].

### Cell viability by crystal violet staining

MCF-7 and MDA-MB-231 cells were seeded into 6-well plates at density of 200,000 cells/well and incubated overnight at 37 °C prior to 2HF treatment. 2HF stock was prepared in DMSO and diluted in cell culture medium, and added to each well at the indicated concentrations. Cell viability was measured 48 h later using crystal violet staining (0.5% (w/v) in 20% methanol). Absorbance was measured using a Tecan microplate reader (Tecan Infinite M200 Pro, Tecan Group Ltd., Männedorf, Switzerland). Cell viability of 2HF-treated cells was expressed as a percentage of control cells (*i.e.* cells treated with equivalent concentrations of the DMSO vehicle). The final concentration of DMSO exposed to the cells was 0.1% (v/v) for the duration of the experiment. Values are presented as mean ± SD from three separate experiments [47].

### Annexin V assay

Apoptosis assays of human breast cancer cells based on loss of membrane integrity were carried out using Annexin V-FITC Apoptosis Detection Kit as described by the supplier (BD Biosciences Pharmingen, San Diego, CA, USA), in which the early- and late-death cells were stained with Annexin V-FITC and PI (propidium iodide). Cells were analyzed using a CyAn ADP cytometer (Beckman Coulter Inc) to quantify fluorescence. Apoptotic cells were defined as Annexin V-FITC positive.

### Detection of apoptotic bodies by TUNEL assay

1x10^5^ cells were grown on the cover slips for ∼12 h followed by treatment with 2HF (50 μM) for 24 h. Apoptosis was determined by the labeling of DNA fragments with terminal deoxynucleotidyl-transferase dUTP nick-end labeling (TUNEL) assay using Promega apoptosis detection system according to the protocol described previously. Slides were analyzed under a fluorescence microscope using a standard fluorescein filter set to view the green fluorescence at 520 nm and red fluorescence of propidium iodide at >620 nm [46].

### *In vitro* migration assay

Cell migration was determined using a scratch assay. 2x10^4^ MCF-7 and MDA-MB-231cells were seeded in 6-well plates to reach 100% confluence within 24 h and then treated with 10 μM mitomycin C for 2 h followed by 25 μM 2HF treatment. Subsequently, a similarly sized scratch was made with a 200 μL pipette tip across the center of each well and immediately imaged at baseline and then at 6h, 12h, and 24 h using an EVOS FLAuto Microscope and Imaging system, Thermo Fisher Scientific, San Diego, CA. The rate of cell migration was determined by comparing the sizes of scratch area using Image J software. Statistically different at *p* < 0.05 when compared with control.

### Flow cytometry analysis of cell cycle regulation

The effect of 2HF on cell cycle distribution was determined by FACS analysis. 2 x 10^5^ cells were treated with 2HF (ranging from 10 -100 μM) for 24 h at 37 ^°^C. After treatment, floating and adherent cells were collected, washed with PBS, and fixed with 70 % ethanol. On the day of flow analysis, cell suspensions were centrifuged; counted and same numbers of cells were resuspended in 500 μl PBS in flow cytometry tubes. Cells were then incubated with 2.5 μl of RNase (stock 20 mg/ml) at 37 ^°^C for 30 min after which they were treated with 10 μl of propidium iodide (stock 1mg/ml) solution and then incubated at room temperature for 30 min in the dark. The stained cells were analyzed using the Beckman Coulter Cytomics FC500, Flow Cytometry Analyzer. Results were processed using CXP2.2 analysis software from Beckman Coulter.

### Immunoblotting

Tumor tissues from control and 25, 50 and 100 mg/kg b.w. 2HF-treated mice were processed for immunoblotting as described by us previously [26]. Supernatant proteins were resolved by sodium-dodecyl sulfate polyacrylamide gel electrophoresis and transferred onto polyvinylidene fluoride membrane. Change in the level of desired protein was determined by densitometric scanning of the immuno-reactive bands. Equal loading of proteins was confirmed by stripping and re-probing the membranes with β-actin antibodies.

### *In-vivo* xenograft studies

Hsd: Athymic 8 wk old female nude nu/nu mice were purchased from Charles River, Wilmington, MA, and acclimated for a week prior to start of the experiment. All animal experiments were carried out in accordance with a protocol approved by the Institutional Animal Care and Use Committee (IACUC). Twenty mice were divided into four groups of 5 animals (treated with vehicle only i.e. corn oil and 2HF at the doses of 0.0025%, 0.005% and 0.01% w/w). Exponentially growing MDA-MB-231 cells were suspended in PBS and mixed in a 1:1 ratio with Matrigel. A 0.1 mL suspension containing 1x10^6^ cells was injected subcutaneously on right flank of each mouse above the hind limb. At the same time, animals were randomized into control and treatment groups. Treatment was started 10 days after the MDA-MB-231 cells implantation to see palpable tumor growth. Treatment consisted of 2HF at the doses of 0.0025%, 0.005% and 0.01% (w/w), equivalent to 25, 50 and 100 mg/kg b. w. respectively, in 200 μl corn oil by oral gavage alternate day. Control groups were treated with 200 μl of corn oil only. Animals were examined daily for signs of tumor growth. Tumors were measured in two dimensions using calipers and body weights were recorded. Each mouse in every group was monitored on alternate days for signs of distress and areas of swelling or redness. Photographs of animals were taken at day 1, day 10, day 20, day 40, and day 60 after subcutaneous injection, are shown for all groups. Photographs of tumors were also taken at day 60.

### Histopathological examination of tumors for angiogenic, proliferative and differentiation markers

Breast tumors (control as well as 25, 50 and 100 mg/kg b.w. 2HF treated) were harvested from mice bearing tumors for 60 d. Tumor samples fixed in buffered formalin for 12 h were processed conventionally for paraffin-embedded tumor sections (5 μm thick). Hematoxylin and Eosin (H&E) staining to assess hyperplasia was performed on paraffin-embedded tumor sections. Histopathologic analyses for protein markers with anti-E cadherin to analyze tumor suppressor/ differentiation effects, anti-CD31 to visualize blood vessels, anti-Ki67 to assess cell proliferation, anti-vimentin to analyze mesenchymal cells, and anti-PCNA to assess proliferating cell nuclear antigen were also performed, using Universal ABC detection kit (Vector). Statistical significance of difference was determined by two-tailed Student’s *t* test. Immuno-reactivity is evident as a dark brown stain, whereas non-reactive areas display only the background color. Sections were counterstained with Hematoxylin (blue). Photomicrographs at 40x magnification were acquired using Olympus DP 72 microscope. Percent staining was determined by measuring positive immuno-reactivity per unit area. The intensity of antigen staining was quantified by digital image analysis using DP2-BSW software. Bars represent mean ± S.E. (n = 5); * *p*<0.001 compared with control.

### Statistical analysis

Each experiment was repeated at least twice to ensure reproducibility of the results. All data were evaluated with a two-tailed unpaired Student’s t test are expressed as the mean + SD. Changes in tumor size and body weight during the course of the experiments were visualized by scatter plot. The statistical significance of differences between control and treatment groups was determined by ANOVA followed by multiple comparison tests. Differences were considered statistically significant when the *p* value was less than 0.05.

## Abbreviations

AKT: protein kinase B (PKB), also known as AKT
BAX: BCL-2-associated X protein
BCL-2: B-cell CLL/lymphoma 2
BIM: also known as BCL2L11
CD 31: cluster of differentiation 31
CDKs: cyclin-dependent kinases
ER: estrogen receptor
ERBB2: Erb-B2 receptor tyrosine kinase 2 or HER2
ERK: extracellular signal-regulated kinase
PR: progesterone receptor
HER2: human epidermal growth factor receptor 2
2HF: 2′-hydroxyflavanone
JAK2: Janus kinase 2
Rb: retinoblastoma
RLIP76: A 76 kDa ral-binding protein, RALBP1 or RLIP76
PCNA: proliferating cell nuclear antigen
PARP: poly ADP ribose polymerase
STAT3: signal transducer and activator of transcription 3
TUNEL: terminal deoxynucleotidyl transferase-mediated dUTP nick-end labeling
VEGF: vascular endothelial growth factor
